# Paternal and maternal long-term psychological outcomes after uterine artery embolization for severe post-partum hemorrhage

**DOI:** 10.1038/s41598-021-92847-z

**Published:** 2021-07-07

**Authors:** Maude Bernasconi, Béatrice Eggel-Hort, Antje Horsch, Yvan Vial, Alban Denys, Thibaud Quibel, David Baud

**Affiliations:** 1 Materno-fetal & Obstetrics Research Unit, Department of Obstetrics and Gynecology, Lausanne University Hospital, 1011 Lausanne, Switzerland; 2 Institute of Higher Education in Healthcare Research, University of Lausanne and Lausanne University Hospital, Lausanne, Switzerland; 3 Neonatology Service, Department Woman-Mother-Child, Lausanne University Hospital, Lausanne, Switzerland; 4 Department of Radiology, Lausanne University Hospital, 1011 Lausanne, Switzerland; 5 Obstetric Service, Department “Femme-Mère-Enfant”-“Woman-Mother-Child”, Centre Hospitalier Universitaire Vaudois (CHUV) & University of Lausanne (UNIL), 1011 Lausanne, Switzerland

**Keywords:** Psychology, Anatomy, Diseases, Risk factors

## Abstract

This study intend to compare the long-term psychological impact (depression, post-traumatic stress disorder) on both partners between patients that underwent uterine artery embolization (UAE) for post-partum hemorrhage (PPH) and uneventful deliveries. Women who experienced severe PPH treated by UAE in our institution between 2003 and 2013 were identified in our obstetrical database. These cases were matched to controls with uneventful deliveries. Matching criteria were maternal age, parity, ethnicity, year of delivery, birthweight, gestational age and mode of delivery. Patients and their partners completed validated questionnaires measuring post-traumatic stress (TSQ), as well as depression symptoms (MINI). A total of 63 cases of PPH and 189 matched controls (1:3) participated in a study exploring gynecological and obstetrical outcomes. With a mean of 8 years post-index delivery, patients after PPH showed increased risk of depression (p = 0.015) and post-traumatic stress disorder (22.2% versus 4.8%, p < 0.005) compared to controls. PPH remains strongly associated with post-traumatic stress disorder, even after adjustment for depression (adjusted odds ratio 5.1; 95% confidence intervals 1.5–17.5). Similarly, partners of patients with PPH showed a propensity to depression (p = 0.029) and post-traumatic stress disorder (11.5% versus 1.5%, p = 0.019). In conclusion, both women and their partners are at increased risk of long-term psychological adverse outcomes after PPH. Couples may benefit from psychological support.

## Introduction

Post-partum hemorrhage (PPH) remains one of the primary causes of maternal death with a mortality rate of 1 per 10,000 deliveries^1^. PPH has been defined as an estimated blood loss (EBL) of 500 mL or more after vaginal delivery or 1000 mL or more after cesarean delivery. First line treatment of PPH includes fundal massage, administration of uterotonic drugs (e.g. oxytocin and sulprostone), active resuscitation, manual exploration of the uterine cavity and surgical repair of genital tract lacerations. Since 1979, Uterine Artery Embolization (UAE) is proposed as an alternative to surgical procedures^2^. It avoids more aggressive surgical management of in-tractable post-partum hemorrhage, such as pelvic vessel ligation, uterine compression suture techniques, hemostatic emergency hysterectomy, and subsequent complications of emergency pelvic surgery. The advantages of UAE are easy identification of bleeding site, decreased re-bleeding from collaterals, as more distal occlusion of bleeding vessels is carried out, preservation of the uterus and thus fertility, avoidance of a laparotomy, and technically difficult hysterectomy.

The efficacy and immediate safety of this conservative technique has been demonstrated, and UAE is now accepted to be a safe and reliable procedure when available. Several reports have described the efficacy, early and long-term maternal complications of UAE for PPH, as well as the impact of these procedures on fertility and future obstetrical outcome^3^.

Besides physical morbidities, PPH may represent a traumatic childbirth experience, which can also cause psychological sequelae such as posttraumatic stress disorder (PTSD) and postpartum depression. However, the psychological impact of PPH has scarcely been studied. A PPH that was sufficiently severe to require a uterine-sparing procedure can involve exposure to actual or threatened death or severe injury of the woman, thus meeting diagnostic criteria for a traumatic stressor^4^. Evidence on the psychological consequences of other obstetric events, such as traumatic childbirth, shows that both women and their partners can develop mental health problems. PTSD consists of four symptom clusters (intrusions, avoidance, hyperarousal and negative cognitions and mood) and can be diagnosed at least 1 month after the traumatic stressor occurred^4^. Prevalence rates of 3–4% in community samples and approximately 16–18% in high-risk samples following childbirth have been reported^5,6^. One study interviewed a cohort of 206 women with PPH, among which 5% and 3% showed evidence of PTSD at two and 4 months post-delivery, respectively^7^. Michelet et al*.*^8^ studied the psychological impact of hysterectomy for life-threatening PPH. They showed that 64% of the patients presented with PTSD after PPH. A total of 50% of the women who underwent an emergency peripartum hysterectomy saw a psychologist after the index hospitalization.

Approximately one in seven mothers is at risk of developing postpartum depression, characterised by feelings of low mood, loss of interest in usual activities, feelings of worthlessness, and loss of energy^9^. The prevalence of postpartum depression is approximately 15% in high income countries^10^. Maternal PTSD and depression are associated with mother-infant relationship problems, as well as negative developmental outcomes of the infant^11^. Early identification of women with symptoms of postpartum PTSD or depression and rapid access to evidence-based treatment where appropriate are therefore vital^12^.

Research on paternal PTSD and depression so far is scarce. Paternal postpartum PTSD prevalence rates in community samples are approximately 5% and between 1–8% for depression^13^. Evidence shows significant relationships between maternal and paternal mental health, although the causality and direction of this relationship often remains unclear^13^.

Our primary aim was to estimate the long-term psychological impact of severe PPH in a large single-center cohort of women and their partner. Our primary outcome was the risk of depression and of PTSD symptoms, investigated through validated questionnaires (respectively MINI and TSQ). These patients were compared to matched controls with uneventful deliveries.

## Materials and methods

The detailed methodology was described in a joined paper investigating gynecological, reproductive and sexual outcomes after UAE for PPH in the same population^14^. Briefly, we conducted a case control study using our obstetrical database of more than 30,000 patients, from the Maternity Unit of the Lausanne University Hospital (Switzerland)^15^. We selected all women who experienced a severe PPH treated by UAE in our institution between 2003 and 2013, regardless of the procedure. Data included maternal age, ethnicity, year of delivery, parity, mode of delivery, birth weight, and gestational age. We excluded deliveries below 24 weeks of gestational age and those with UAE that required hysterectomy.

In our institution, UEA is available 24/7, and was considered at this period the principal second line procedure when initial first line procedures for PPH have failed to stop an active bleeding both after a vaginal or caesarean delivery. The PPH treated with UAE cases were matched (ratio 1:3) with patients with uneventful deliveries (without PPH/UAE), with consideration of maternal age, ethnicity, year of delivery, parity, mode of delivery, birth weight and gestational age. Three controls, who delivered just after each case, were chosen for each case.

Our primary aim was to estimate the long-term psychological impact of severe PPH in a large single-center cohort of women and their partner. Our primary outcome was the risk of depression and of PTSD symptoms, investigated through validated questionnaires (respectively MINI and TSQ). gynecological symptoms, sexuality, future fertility, and obstetrical outcomes were investigated in a previous study^14^. The study was approved by the Ethical Committee of the University and Hospital, Lausanne, Switzerland (protocol 55/13; date of approval 07.16.2013). We confirm that all methods were performed in accordance with the relevant guidelines and regulations, and informed consent was obtained from all participants.

To assess the psychological impact of PPH, women were asked whether they consented to complete 2 validated questionnaires. Symptoms of depression were assessed with the “Mini International Neuropsychiatric Interview” (MINI)^16^ and symptoms of PTSD with the Trauma Screening Questionnaire (TSQ)^17^. The same questionnaires were also offered to their partners. Non-responders received the questionnaires a second time by mail. Data on current social and demographic characteristics of the patients were also collected by self-report.

The MINI is a structured screening interview developed in France and the United States for DSM-IV and ICD-10 psychiatric disorders^16^. It is structured to allow administration by non-specialized interviewers and focuses on the existence of current disorders. For each disorder, one or two screening questions rule out the diagnosis when answered negatively. The MINI is short (15 min), validated in French, has high reliability and validity^17,18^, and is based on questions commonly asked by the physician, making it “user friendly.”

The TSQ^19^, also validated in French, is a 10-item self-reporting measure that was designed for use with survivors of all types of traumatic stress^20^ and is a screening instrument for PTSD symptoms (upsetting memories, recurring dreams, impression of re-experiencing the event, feeling upset at reminders of the event, bodily reactions, sleep disorders, irritability, difficulty concentrating, hypervigilance, and being jumpy or startled) derived from the PTSD symptom scale self-report version (PSS-SR). The total questionnaire score (i.e., the sum of the items) ranged from zero to 10. Each item is derived from the DSM-IV criteria and describes either a re-experiencing symptom of PTSD (items 1–5) or an arousal symptom of PTSD (items 6–10). Respondents are asked to endorse those items that they have experienced at least twice in the past week. A TSQ score ≥ 7 was considered as PTSD^21^.

Regarding the sample size calculation, Grekin et al.^5^ demonstrated that the prevalence of postpartum PTSD in community samples was estimated to be 3.1% and approximately 16–18% in high-risk samples. On the basis of these estimates, we determined that a sample size of 57 cases and 171 controls (ratio 1:3) would have an 80% power to detect a similar difference with a significance level of 0.05.

### Data analysis

The demographic data and results of the questionnaires were compared between patients and/or their partner in cases with and without PPH by the Pearson χ^2^ test (or the Fisher’s exact test when indicated) for categorical variables. For continuous variables, means were compared by the Student’s t test (when normally distributed) or the Wilcoxon test (when non-normally distributed). Statistical analyses were performed using STATA-15 (Stata Corporation, College Station, USA).

## Results

From 2003 to 2013, 77 patients were treated by UAE for PPH, of which 12 were lost to follow-up and 2 declined participation in the study. A total of 63 cases (81.8%) were ultimately included in our study. Three matched controls (n = 189) were chosen for each case (n = 63)^14^.

As shown in our previous paper^14^ describing the same cohort of patients, maternal age (33 years old), gestational age at birth (38 weeks), parity, birth weight, arterial pH, Apgar at 5 min were all similar between cases and controls.

Among the 252 women contacted by phone, 142 (56%) agreed to also complete questionnaires investigating the psychological impact of their delivery. Demographic and obstetrical characteristics of responders were similar to those of non-responders and of patients lost to follow-up (data not shown). A total of 38 women that underwent UAE and 26 of their partners completed and returned the questionnaires by post. Additionally, 104 controls and 66 of their partners did the same. The response rates were similar between patients; questionnaires from 38/63 (60%) and 104/189 (55%) women with and without PPH, respectively, were returned (p = 0.56). With respect to their partners, 92 partners completed the questionnaires; 26/63 (41%) and 66/189 (35%) partners from patients with or without PPH, respectively (p = 0.37). The mean interval between the index delivery and response to the questionnaires was 8.1 years (95% confidence intervals [95%CI] 7.8–8.4)^14^. General socio-demographic variables were similar between both groups^14^.

Compared to the control patients with uneventful deliveries, patients after PPH showed an increased risk of symptoms of depression (Table 1, MINI score p = 0.015). Women with PPH were more likely than controls to have a depressed mood (p = 0.043), to lose interest in most things (p = 0.05), to have motor disorders (excitation or slowing) (p = 0.003) and to be tired (p = 0.047). While depression in general was more likely in women after PPH, there were no statistically significant differences in specific symptoms, such as appetite, weight, sleep quality, feelings of guilt and loss of concentration between the two groups (Table 1).

**Table 1:** Maternal psychological symptoms.

MATERNAL answers		Patients WITHOUT embolization % (n = 104)	Patients WITH embolization % (n = 38)	p values
**MINI questionnaire**				
1	Depressed mood	26.9	44.7	0.043
2	Less interested in most things	_21.1_	23.7	0.747
3	Much less interested in most things	31.7	57.9	0.005
4	Change in appetite or weight	31.7	39.5	0.388
5	Sleeping disorders	30.1	39.5	0.292
6	Being slowed or excited	26.0	52.6	0.003
7	Being tired	42.7	68.4	0.007
8	Feel worthless?	23.8	29.7	0.475
9	Feel guilty	27.5	36.8	0.281
10	Inability to concentrate or make decisions	23.5	39.5	0.061
	Score MINI (1–10)	2.7 ± 0.3	4.1 ± 0.5	0.015
**TSQ questionnaire**				
1	Upsetting thoughts or memories	16.4	55.3	< 0.001
2	Upsetting dreams	7.7	21′1	0.026
3	Acting as tough the event were happening again	1.9	13.2	0.006
4	Feeling upset by reminders	17.3	57.9	< 0.001
5	Bodily reactions	24.3	31.6	0.382
6	Sleeping disorders	24.3	31.6	0.382
7	Irritability or anger	40.8	44.8	0.672
8	Difficulty concentrating	27.2	36.9	0.266
9	Heightened awareness	42.0	47.4	0.570
10	Feeling jumpy	29.1	34.2	0.714
	Score TSQ (1–10)	2.0 ± 0.2	3.5 ± 0.4	< 0.001

Concerning PTSD symptoms (TSQ, Table 1), PPH patients suffered more frequent upsetting thoughts or memories (p < 0.001), acted more as if the event were happening again (p = 0.006) and were more likely to be upset by reminders (p < 0.001). These patients showed an increased risk of PTSD (p < 0.001) compared to controls. Upsetting dreams, somatic reactions, sleeping disorders, mood disorders (irritability or anger), inability to concentrate, heightened awareness, and an increased startle response were also more prevalent after PPH but did not reach statistical significance (Table 1). Respectively 22.2% and 4.8% of women with and without PPH reached the definition of PTSD (p < 0.005). PPH remains strongly associated with post-traumatic stress disorder, even after adjustment for maternal depression (adjusted odds ratio 5.1; 95% confidence intervals 1.5–17.5).

Partners from patients with previous PPH showed a propensity towards depression (Table 2, MINI score, p = 0.029) and PTSD (Table 2, TSQ score, p = 0.019) compared to the control partners. More specifically, partners from patients with PPH reported significantly “less interest in most things” (Table 2, MINI, p = 0.024), more “change in appetite or weight” (Table 2, MINI, p = 0.002), “upsetting thoughts or memories” (Table 2, TSQ, p = 0.011) or “feeling upset by reminders” (Table 2, TSQ, p = 0.002) than the control partners. Respectively 11.5% and 1.5% of partners of women with and without PPH reached the definition of PTSD (p = 0.036).

**Table 2:** Paternal psychological symptoms.

PATERNAL answers		Partners of patients		P values
		WITHOUT embolization % (n = 104)	WITH embolization % (n = 38)	
**MINI questionnaire**				
1	Depressed mood	6.2	19.2	0.059
_2_	Less interested in most things	_4.6_	_15.4_	_0.082_
3	Much less interested in most things	4.6	19.2	0.024
4	Change in appetite or weight	6.1	30.8	0.002
5	Sleeping disorders	9.1	23.1	0.075
6	Being slowed or excited	9.1	7.7	0.830
7	Being tired	12.1	15.4	0.676
8	Feel worthless?	3.0	7.7	0.323
9	Feel guilty	13.6	15.4	0.828
10	Inability to concentrate or make decisions	4.6	15.4	0.077
	Score MINI (1–10)	0.7 ± 0.2	1.7 ± 0.5	0.029
**TSQ questionnaire**				
1	Upsetting thoughts or memories	16.9	42.3	0.011
2	Upsetting dreams	4.6	3.9	0.872
3	Acting as tough the event were happening again	1.5	7.7	0.137
4	Feeling upset by reminders	12.7	42.3	0.002
5	Bodily reactions	6.3	11.5	0.396
6	Sleeping disorders	10.9	19.2	0.294
7	Irritability or anger	10.9	23.1	0.138
8	Difficulty concentrating	17.2	15.4	0.835
9	Heightened awareness	20.6	26.9	0.518
10	Feeling jumpy	4.7	15.4	0.086
	Score TSQ (1–10)	1.0 ± 0.2	2.1 ± 0.5	0.019

## Discussion

This study investigated the long-term (8 years post-delivery) psychological impact of severe PPH in a large single-center cohort of women requiring UAE. Our data suggest that compared to patients with uneventful deliveries, patients with PPH show increased risk of depression (MINI) and of PTSD (TSQ) symptoms. Partners from patients with previous PPH showed a similar pattern, with higher risk for depression and PTSD symptoms than control partners. We demonstrate that PPH can be experienced as a traumatic event for both the mother and her partner.

Despite patients were selected according to their UAE status, the main factor of comparison is the occurrence or not of severe PPH and not the procedure performed. If we would have investigated the psychological impact of different PPH treatments, groups of severe PPH who received conservative medication treatment, conservative surgical procedures and hysterectomy would need to be compared. Thus, among women who had the same complication, the different forms of treatment could be compared as greater or lesser related to the occurrence of PTSD or depression. However, the goal of the present study was to investigate the impact of PPH and not the treatment option.

Grekin et al*.*^5^ demonstrated that the prevalence of postpartum PTSD in community samples was estimated to be 3.1% and approximately 16–18% in high-risk samples. Important risk factors in community samples included current depression, labor experiences, as well as a history of psychopathology. Yildiz et al*.*^6^ and Cook et al*.*^11^ measured postpartum PTSD with a mean prevalence of 4%. In their study, Thompson et al*.*^7^ demonstrated that after a PPH, 5% of the patients showed evidence of PTSD at 2 months after childbirth and 3% at 4 months. Wisner et al*.*^9^ showed that 14.5% of new mothers experience depressive episodes that impair maternal role function. For Gelaye et al*.*^10^, the prevalence of postpartum depression among women residing in high-income countries is reported to be 10% and 20% for women in low income and middle-income countries.

The “COMMAG cohort study”^22^ performed a multidimensional assessment of women who experienced severe maternal morbidity and its short-term and medium-term impact on the lives and health of women and their children. The exposed population was selected from intensive care unit admissions of one hospital in Brazil, whereas controls were randomly selected among women without severe morbidity. Altered PTSD had a similar prevalence between groups. A possible explanation mentioned by the authors to explain this result was the time elapsed between the event and the interview ranging (6 months to 5 years). In contrast to our study, many different pathologies were grouped under “severe maternal morbidity”, with probably a significant number of patients who were intubated or sedated in intensive care unit. Angelini et al.^23^ also evaluated women with or without severe maternal morbidity, and found no association with PTSD 5 years after birth. In our study, one of the criteria to perform UAE was that the patients remain stable hemodynamically and thus conscious of the ongoing traumatic situation. Also, PPH appear suddenly just after birth mainly in patients without risk factors, in contrast to severe preeclampsia or severe maternal comorbidities which often begin before birth.

In a systematic review evaluating the relationship between severe maternal morbidity and PSTD, Furuta et al*.* found inconstant evidence of this relationship, notably when women had experienced major obstetrical haemorrhage (blood loss > 1500/2500 ml or transfused with 5 or more units of blood)^24^. Nevertheless, most of the studies reported in this review used different tools to investigate PTSD and were based on a short interval time between evaluation and the traumatic event. Persistence of depression and PTSD during longer period of time have less been studied. However, their consequences on infant/childhood development are significant^25, 26^.

In our study, women who had PPH experienced significantly more upsetting feelings or thoughts than women whose delivery was uneventful. They had more reminiscence of this event, as they noted more frequently acting as though the event was coming again. Sentilhes et al*.*^27^ also evaluated the long term psychological impact of severe PPH (mean follow up of 7.1 years) by asking women about their experience and perceptions of PPH. With a non-validated structured interview, 7.3% reported persistent fear of dying, and consequences on their sexual and marital life. In our study, when information regarding the reason behind no partner response was available, the most frequent explanation was that the couple was separated, often shortly after childbirth.

Of note, Eggel et al*.*^14^ demonstrated that when patients with and without PPH were asked whether they were considering a subsequent pregnancy after the index pregnancy, the rate of positive responses was similar between both groups. For those who did not want another pregnancy, however, “fear as a reason not to get pregnant” was significantly higher in patients who had PPH. Patients with PPH took significantly more time to conceive after than those without PPH (35 versus 18 months, p = 0.002). The arguments put forward by patients to justify this prolonged interval were variable, but the fear of a recurrent PPH was again raised in this situation.

Partners who witnessed a severe PPH might feel very distressed and helpless during a procedure which can last hours, notably if the patient is transferred to another unit or another hospital. In a prospective Australian study^28^, partners of women whose delivery was complicated by severe PPH reported higher scores than control partners on symptoms of posttraumatic stress, even though none was diagnosed with PTSD.

The strengths of our study were the use of validated questionnaires assessing the psychological impact of PPH, the presence of a control group with similar sociodemographic and obstetrical characteristics, and inclusion of the partners. So far, only one qualitative study, involving 11 women and 6 partners, has investigated the psychological impact of PPH on both members of the couple^29^.

One limitation of the present study was its retrospective design which prevents us from drawing causal conclusions. Indeed, we were unable to describe all the cofounding factors that could potentially influence our results, notably if patients and their patrners had previous psychiatric disorders. Also, as many patients still present depressive symptoms or PTSD, we could imagine that some of them had gotten psychological help or treatment during the interval between the index pregnancy and our study. Another limitation was the lower response rate for the partners compared to the patients. Fewer partners from patients with uneventful deliveries answered (63.5%) than partners from PPH cases (68.4%), without reaching significance (p = 0.584). We might hypothesize that non-responder partners feel less involved in the delivery events, especially when the latter is uneventful. When information regarding the reason behind no partner response was available, the most frequent explanation was that the couple was separated, often shortly after childbirth. Another limitation of the study results is the fact that several years passed since the index delivery (8 years), potentially inducing a recall bias.

Our results show that PPH can be experienced as a traumatic event by both mothers and their partners, which may lead to the development of PTSD and prolonged depression symptoms. It may therefore be helpful if care providers considered screening for these mental health difficulties. Postpartum follow-up appointments with the couple and healthcare staff present during childbirth should be proposed to provide information regarding the events that occurred during childbirth and the decisions taken by the healthcare team. This would also allow the couple to ask questions they may have and to evaluate their need for psychological support.

## Abbreviations

UAE: Uterine arterial embolization
PPH: Postpartum hemorrhage
MINI: Mini International Neuropsychiatric Interview
TSQ: Trauma Screening Questionnaire
PTSD: Post-traumatic stress disorder
